# Advances and Challenges in 3D Bioprinted Cancer Models: Opportunities for Personalized Medicine and Tissue Engineering

**DOI:** 10.3390/polym17070948

**Published:** 2025-03-31

**Authors:** Sai Liu, Pan Jin

**Affiliations:** Health Science Center, Yangtze University, Jingzhou 434023, China; jinpanflyman@163.com

**Keywords:** 3D bioprinting, tumor model, tumor microenvironment, personalized medicine

## Abstract

Cancer is the second leading cause of death worldwide, after cardiovascular disease, claiming not only a staggering number of lives but also causing considerable health and economic devastation, particularly in less-developed countries. Therapeutic interventions are impeded by differences in patient-to-patient responses to anti-cancer drugs. A personalized medicine approach is crucial for treating specific patient groups and includes using molecular and genetic screens to find appropriate stratifications of patients who will respond (and those who will not) to treatment regimens. However, information on which risk stratification method can be used to hone in on cancer types and patients who will be likely responders to a specific anti-cancer agent remains elusive for most cancers. Novel developments in 3D bioprinting technology have been widely applied to recreate relevant bioengineered tumor organotypic structures capable of mimicking the human tissue and microenvironment or adequate drug responses in high-throughput screening settings. Parts are autogenously printed in the form of 3D bioengineered tissues using a computer-aided design concept where multiple layers include different cell types and compatible biomaterials to build specific configurations. Patient-derived cancer and stromal cells, together with genetic material, extracellular matrix proteins, and growth factors, are used to create bioprinted cancer models that provide a possible platform for the screening of new personalized therapies in advance. Both natural and synthetic biopolymers have been used to encourage the growth of cells and biological materials in personalized tumor models/implants. These models may facilitate physiologically relevant cell–cell and cell–matrix interactions with 3D heterogeneity resembling real tumors.

## 1. Introduction

**Figure 1:**
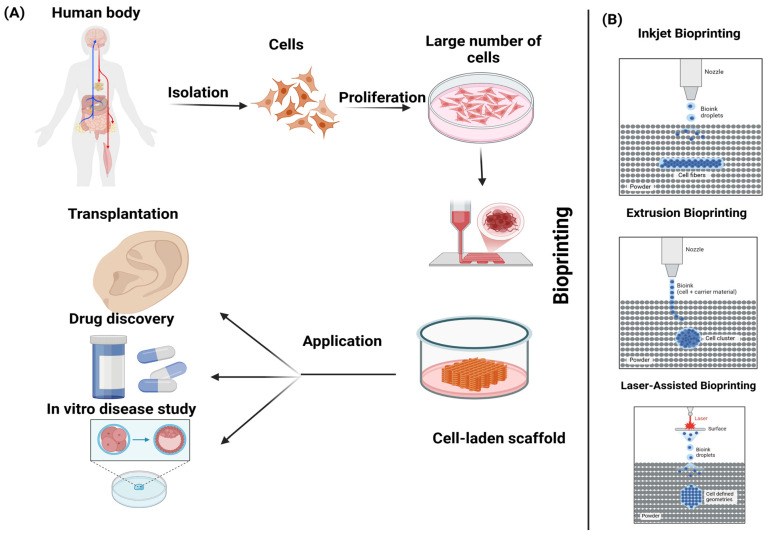
(**A**) The typical bioprinting workflow for therapeutic applications involves isolating and expanding human cells, printing cell-laden scaffolds, and using these scaffolds for therapy, drug testing, or disease modeling. (**B**) Three types of bioprinters. Created in https://BioRender.com.

Globally, cancer has emerged as the predominant cause of mortality, accounting for nearly one-sixth of all deaths in 2020. The urgency to elucidate cancer’s developmental mechanisms to discover more efficacious treatments is underscored by this mortality rate. The genesis and evolution of cancer are intricately linked to the heterogeneity of cancer cells and their dynamic interplay with the surrounding microenvironment. The conventional 2D culture model, characterized by a homogenous cell type and equalized nutrient and growth factor distribution, fails to capture the complexity of cellular interactions and intratumoral diversity. Consequently, data derived from 2D models diverge significantly from in vivo conditions [1,2]. Although in vivo tumor models utilizing experimental animals are prevalent, disparities in genetics and species result in data that may not accurately mirror human tumorigenesis and progression. Additionally, these models are encumbered by ethical considerations and necessitate extensive cultivation periods, rendering them both time-intensive and laborious [3]. To reconcile the discrepancies between 2D cultures and animal models, 3D culture models have been innovated [1]. The advent of 3D printing technology has facilitated its application within the biomedical domain. These models adeptly emulate the in vivo microenvironment and biomimetic human tissue architecture, thereby enabling the exploration of cancer initiation and progression mechanisms. Furthermore, they hold potential for clinical utility in precise surgical navigation, among other applications [4,5,6]. This review accentuates the pivotal role of 3D cancer models in reconstructing the tumor microenvironment and their prospective clinical implementations while also acknowledging the existing challenges (Figure 1).

## 2. Technical Methods of 3D Printing

**Figure 2:**
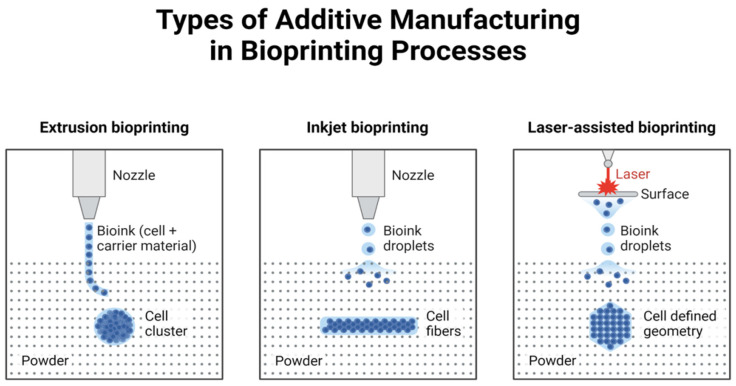
The figure illustrates key 3D bioprinting methods: laser-assisted techniques (LIFT and LIBP), photopolymerization methods (TPP and DLP), and extrusion-based methods.

Bioprinting technology mainly utilizes laser printing, inkjet printing, and extrusion [7]. The combination of cells and biomaterials is used to form layer by layer based on the CAD model of tissues and organs. It differs from the traditional composite manufacturing way of “cells and scaffolds”, providing accurate spatial positioning for the cells. Multiplexed 3D printing of cells: multiple types of living cells or biological materials can be printed simultaneously (e.g., muscle and endothelial cells) [8] and create functional vascularized tissue (Figure 2).

### 2.1. Inkjet Bioprinting

Inkjet bioprinting technology has matured since the beginning of the twentieth century. It is the earliest application of biological printing technology. It is a non-contact technology further classified into ink (cells or biological material) 3D printing, which fabricates organs on the basis of a computerized digital model of tissues and organs [9,10].

In thermal inkjet printing technology, a heating element is commonly used to vaporize the droplets. The element is utilized to swiftly heat the area surrounding the ink, causing the ink in the pressure cavity to vaporize into bubbles [11]. The gasification pressure causes the bioink to leak from the extrusion nozzle as the surface tension of the droplet is overcome. When the voltage stops being applied, the cooling phase occurs quite rapidly, within which time it all boils back into space. The voltage can be applied and released many times, squeezing the droplets continuously. This type of droplet is mostly used to spray piezoelectric materials in piezoelectric inkjet printing technology. When voltage is applied to both ends of the piezoelectric element, the piezoelectric material deflects and squeezes out the droplet near the nozzle. The piezoelectric element returns to its original shape when the voltage is released [12]. This method enables continuous extrusion of droplets by repeatedly applying and releasing voltage. According to Cui et al. [13], inkjet printing technology can be used to regenerate the articular cartilage of the human body, demonstrating a promising ability to guide tissues to efficiently recover. A system controls the process parameters, such as cell concentration, the volume and precision of the droplet, nozzle diameter, and average diameter for printing cells and biological materials [14]. A multi-nozzles inkjet printing platform for the fabrication of composite structures has been developed by Weiss et al. [15,16]. Different growth factors (fibrinogen, thrombin) and cells were printed to treat skull defects in mice in situ. They demonstrated its in situ printability; however, it was not practical for use due to its complexity.

In comparison, inkjet printing technology shines due to its long history and relative maturity, with the main advantages including color printing, (1) such that multiple nozzles can be incorporated in synchronization, enabling cell/growth factor/biomaterial co-printing, hence facilitating the generation of heterogeneous tissues/organs [17]. (2) Inkjet printing is a non-contact approach to biofabrication. The nozzles and the culture medium are also kept separately to minimize possible cross-contamination during printing [18]. Printing can be performed on solid, hydrogel, and liquid interfaces. There is no additional requirement for printing graphic smoothness, which makes in situ printing convenient. (3) Inkjet printing is fast and efficient, leading to high production of organs such as the liver. Additionally, long-term assay-related problems can be solved due to a decline in time-consuming biological activity, making it suitable for large auto part processing. (4) The droplet volume is small, comparable to a single body cell, enabling precise manipulation at the cellular level [19]. Although there are significant research breakthroughs with inkjet printing technology, there are also certain limitations: (1) the small nozzle diameter increases the likelihood of cell precipitation and accumulation, hence restricting the printing density (<5 × 10^6^ cells/mL) [20]; (2) during the heat-induced printing procedure, the nozzle is heated to an elevated temperature that can be detrimental to cells, while the presence of shear stress concurrently diminishes cellular activity; (3) the amalgamation of droplets is challenging, as the morphology of the droplets cannot be precisely regulated. The structural integrity of printed materials is a challenge that must be addressed in inkjet printing.

### 2.2. Extrusion Bioprinting

The extrusion deposition technique secures the print head on a three-axis electronically regulated moving platform. The printing material is extruded from the print head with applied pressure, while the activation and deactivation of the print head are regulated by an on–off valve. The printing material comprises suspended cells combined with extracellular matrix-mimicking substances like agar and hydrogels. Upon extrusion onto the base plate, the printing material undergoes crosslinking to create a superposition, facilitating the formation of a 3D cellular structure [21]. A comparable technique employs a multi-jet extrusion method, which constructs layer by layer by alternating between a layer of heat-sensitive material and a layer of cells, then regulating the temperature to destroy the heat-sensitive material and retain just the cells, thereby creating a three-dimensional cellular structure. The extrusion deposition technique must satisfy the following criteria [22]: (i) The printed material should retain its shape as much as possible after deposition and undergo in situ cross-linking; (ii) the material and printing technique must exert no or minimal influence on the cellular state; (iii) the degrading properties of the scaffolds post-molding must be regulated. Extrusion-based 3D printing techniques are among the most prevalent manufacturing methods utilized in tissue engineering applications [23]. The purpose of extrusion additives is to substitute the ink with a liquid or molten substance that is dispensed through the nozzle, forming a line that solidifies on the stencil. The nozzle adheres to a pre-established trajectory defined by the computer model to fabricate the three-dimensional product incrementally as the material is extruded. The ink is pulled into a heated die, melted in the nozzle, and extruded into various shapes. These materials, typically polymers, can generate filaments exhibiting a solid-to-melt transformation. This method is distinguished by a minimal threshold for scientific processes and a brief duration necessary for platform construction. The precision and viability of printing primarily rely on the printing substance [24]. On the one hand, the deformation of the printing material during extrusion and descent constrains the final molding reliability, resulting in a printing precision in the order of hundreds of microns. On the other hand, the nozzles employed in this technology are prone to obstruction, hence diminishing processing efficiency. The present investigation emphasizes a balance between the biocompatibility and printability of printed materials [25]. examined a prospective application of improved formulations in 3D bioprinting. The bioink mixture was transformed into a semi-solid viscous substance via the twin-screw casting method. The extruded material was subsequently utilized to produce 3D printable bioinks in the presence of sodium alginate polymer [26]. This technology offers advantages over conventional methods by enabling the production of reproducible, customizable, and functional structures, including the 3D printing of circular thin alginate films, thereby facilitating the regeneration of various tissues.

### 2.3. Laser-Assisted Printing

Laser-assisted 3D bioprinting is a bioprinting approach that utilizes laser energy for the artificial development of tissues. The system has three essential types of machinery: (i) a pulsed laser source, (ii) a target for printing biological material, and (iii) a substrate to collect the printed material. This domain of bioengineering technology integrates laser science with biological engineering, aiming to fabricate living tissues and organs using bioink droplets or cells. 3D bioprinting is a swiftly evolving domain garnering significant attention from multiple scientific disciplines. Laser-assisted 3D bioprinting provides an alternative to traditional non-contact methods, including inkjet printing and nozzle-based bioprinting techniques. Laser-assisted 3D bioprinting employs lasers, offering numerous advantages such as high processing precision, non-contact fabrication, simplicity, and cost-effectiveness [27,28].

Laser-assisted 3D bioprinting offers significant advantages in the fabrication of cancer models due to its high precision and ability to construct complex microscale structures. Techniques such as Direct Laser Writing (DLW) and Laser-Induced Forward Transfer (LIFT) allow for the creation of models that closely mimic the intricate tumor microenvironment. For instance, these techniques enable the printing of bioinks containing cancer cells, extracellular matrix components, and growth factors, facilitating the study of cancer progression and drug responses. Moreover, laser bioprinting can accommodate bioresorbable and biocompatible materials, such as fibrinogen and collagen, making it a valuable tool for producing physiologically relevant cancer models [29,30,31].

By employing laser-induced vapor bubbles or direct photopolymerization, these methods achieve high spatial resolution (up to 100 nm in DLW), crucial for replicating the tumor’s cellular heterogeneity. Furthermore, laser-assisted techniques enable the integration of pre-formed cell spheroids, allowing for rapid construction of three-dimensional tumor structures. This capability is particularly important in studying the interaction between cancer cells and the tumor microenvironment, as well as in screening therapeutic interventions [29,30,32].

#### 2.3.1. Direct Laser Writing (DLW)

Direct Laser Writing (DLW) is a laser-assisted bioprinting technique that uses focused laser beams to achieve photopolymerization in photosensitive bioinks. This method offers exceptional resolution (up to 100 nm), making it particularly suitable for replicating the intricate features of the tumor microenvironment. Bioresorbable materials such as gelatin methacrylate (GelMA) and collagen have been successfully employed in DLW to create scaffolds that support cell adhesion, proliferation, and differentiation, which are critical for cancer research applications [33,34].

**Advantages:**

- **High Precision**: DLW enables the construction of microvascular networks, crucial for mimicking tumor angiogenesis.
- **Customizability**: The technique allows the incorporation of multiple bioinks, including cancer cell-laden hydrogels, extracellular matrix proteins, and growth factors, to create physiologically relevant models.
- **Flexibility**: It supports the integration of pre-formed cancer cell spheroids, allowing for rapid assembly of complex tumor structures.

**Challenges:**

- **Material Limitations**: The need for photosensitive bioinks restricts the range of compatible materials.
- **Throughput**: The high resolution of DLW comes at the cost of slower fabrication times, making it less suitable for large-scale models.
- **Cost**: The equipment and processing requirements for DLW are more expensive than alternative bioprinting methods, such as extrusion printing.

DLW’s precision and adaptability make it a valuable tool for studying the dynamics of cancer cell invasion, tumor–stroma interactions, and therapeutic responses. However, its scalability and material limitations necessitate further advancements to fully realize its potential in cancer model development.

#### 2.3.2. Laser-Induced Forward Transfer (LIFT)

Laser-induced forward transfer (LIFT) is a non-contact bioprinting technique that uses a pulsed laser to transfer biological materials, such as living cells, bioinks, or cell spheroids, from a donor substrate to a receiving substrate. This method involves applying a thin layer of bioink or cell-laden hydrogel to a transparent donor substrate, followed by the focused application of a laser pulse. The laser energy generates a localized pressure wave or vapor bubble beneath the material, propelling a droplet or cell-laden jet onto the receiving substrate [35,36].

**Advantages:**

- **Cell Viability**: LIFT has been shown to maintain high cell viability due to its non-contact nature and precise energy control.
- **Resolution**: The technique allows for the deposition of droplets with diameters as small as a few microns, enabling the creation of fine structures and intricate tissue architectures.
- **Compatibility**: LIFT can accommodate various bioinks, including those containing fragile living cells, making it ideal for cancer models requiring physiological accuracy.

**Challenges:**

- **Thermal Effects**: While the energy used in LIFT is finely tuned, excessive laser intensity can generate heat, potentially compromising cell viability.
- **Material Transfer Limitations**: The uniformity and reproducibility of material transfer depend on the bioink’s viscosity and the laser’s energy settings.

This method has been widely employed in cancer research for creating tumor models that replicate the cellular heterogeneity of tumors and their microenvironments. LIFT enables the printing of pre-formed cancer cell spheroids, expediting the development of 3D tumor constructs for drug screening and therapeutic testing [27,35,37,38,39].

The print speed value of 100–1600 mm/s represents the horizontal travel speed of the print head in laser-assisted bioprinting processes. This metric is important in comparing bioprinting techniques, as higher speeds can enhance throughput but may compromise resolution and precision. For example, in Laser-Induced Forward Transfer (LIFT), faster print speeds are suitable for high-throughput applications like large-scale tissue models, whereas slower speeds may be preferred for intricate, high-resolution constructs such as microvascular networks. This value provides insight into the method’s balance between speed, precision, and its suitability for specific applications, such as fabricating cancer models with precise spatial cell arrangements.

#### 2.3.3. Laser-Induced Side Transfer (LIST)

Laser-Induced Side Transfer (LIST), a new and modified type of laser-assisted bioprinting, supports lateral transfer of bioink, contrasting with LIFT, which is based on vertical material ejection. In LIST, a consistent lateral displacement of liquid bioink or particles is created with a laser pulse on a donor substrate. The introduction of such a lateral arrangement provides greater precision and control over the flow and deposition of materials, which makes LIST especially valuable in generating complex tissue architecture and tailorable extensions [40,41,42].

Unlike classical vertical approaches, the LIST uses a modified bioink that enables imaging prior to transfer and avoids direct light interaction. It enables a different set of edits and optimizations to be performed that is not transferable directly from LIFT. This technique has produced high lateral and depth resolution by means of a galvanometric scanning head, allowing fine steering of the laser beam; hence, LIST is ideal for high-resolution bioprinting, such as the manufacture of 3D tissue scaffolds and multicellular structures [43,44].

The system is versatile and works with a range of bioinks, including hydrogels and particulate-based solutions, which, when endowed with energy upon laser-induced particle energy absorbance, can turn from a solid to a fluid state. Bioink composition, laser wavelength, and energy parameters are among several factors affecting the quality and resolution of printed structures. Nanosecond lasers in the UV range and femtosecond lasers in the visible range are generally applied for LIST. The type of laser chosen will depend on the desired resolution and the material and its properties. Micromachining with UV nanosecond lasers, which offers accurate delivery of energy, is capable of enabling material transfer over large distances. On the other hand, femtosecond lasers in the visible spectrum can be focused on the order of ~0.5 microns laterally, making these systems better suited for microscale applications [45,46].

LIST’s transfer speed outperforms other non-continuous printing techniques, making LIST particularly suitable for the high-throughput production of micron-sized structures. For instance, while preserving their integrity and resolution, LIST can print constructs of over 60 mm in 30 seconds. Thus, biocompatible and high-precision tissue models can be produced by LIST, in which the increasing use of hydrogels and cells as bioinks is a recent priority. These advances have special implications in cancer studies, as LIST can be used to construct complex models of tumor microenvironments that more closely represent native tissue cell–cell interactions [39,47].

#### 2.3.4. Laser-Induced Bubble Printing (LIBP)

Laser-Induced Bubble Printing (LIBP) is a novel bioprinting method that utilizes pulsed laser energy to generate vapor bubbles that can be leveraged to eject bioinks and cell-laden hydrogels. This process utilizes the creation of microbubbles in the bioink layer that can be controlled, producing localized pressure that forces the material into the receiving substrate. In particular, its ability to reach a high spatial resolution, together with the droplet size and fine level of placement control, makes LIBP an attractive technology for complex tissue architecture fabrication [48,49].

The LIBP method exhibits versatility in that it can interface with a variety of bioinks (hydrogels, living cells, synthetic biomaterials), which makes it useful for a wide range of biomedical applications. LIBP has also been used in tissue engineering to create 3D scaffolds with spatially defined cell populations. Using this technique, it is also possible to quickly transfer pre-formed cell spheroids or cellular aggregates, paving the way for physiologically relevant tumor microenvironments for cancer studies. The controlled placement of bioinks using LIBP allows for generating reproducible and scalable models [50].

**Advantages:**

- **Easy coating mechanism:** Due to the high deposition rate, LIBP can achieve excellent morphology from just a few seconds of exposure compared to the other laser-assisted post-processing methods.
- **Surface Structures**: Materials can be accurately patterned with precise control over the size and location of vapor bubbles.
- **Cost Efficiency:** Reducing material waste, especially with costly bioinks or cell-laden hydrogels.

**Challenges**:

- **Thermal Effects:** The laser energy required to form bubbles might induce heat that could impact the viability of the cells. Laser parameter tuning should be conducted carefully to eliminate thermal damage.
- **Bubble Dynamics:** Unrestricted growth of bubbles can impact flow uniformity or destabilize transferred materials, imposing difficulties for intricate structures [39,51,52,53,54].

The configuration of laser-assisted bioprinting consists of a nozzle-free system that integrates a near-infrared pulsed laser with a focusing mechanism utilizing a scanning mirror that directs the laser beam onto the biological material [55]. According to the CAD modeling, laser pulses are concentrated on the target area to produce a high-pressure vapor pocket, resulting in the creation of a cell-laden droplet that descends into the receiving substrate. This technique inhibits cell obstruction and preserves cell viability. Its enhanced high-throughput capacity and reproducibility enable the generation of 3D-printed pre-cancerous and cancer models. Hakobyan et al. [56] created replicable 3D arrays of cellular spheroids comprising acinar and ductal exocrine pancreatic cells using laser-assisted bioprinting, which can serve as a 3D model for investigating the early phases of pancreatic ductal adenocarcinoma development. Laser-assisted bioprinting (LAB) was employed to create corneal tissue analogs utilizing human embryonic stem cell (hESC)-derived limbal epithelial stem cells, lamellar corneal stroma with interspersed acellular bioink layers, and layers containing human adipose tissue-derived stem cells (ADSCs). Post-printing, the 3D constructions demonstrated favorable vitality for adipose stem cells, and the epithelial cells were arranged comparable to native corneal stroma, exhibiting migratory potential. The research demonstrated the successful development of multilayer 3D-bioprinted tissues that replicate corneal tissue [57]. Another research has shown that LAB facilitates the printing and organization of nano-hydroxyapatite and human osteoprogenitors while maintaining viability and proliferation for up to 15 days. Additionally, it was proposed as an essential approach for two-dimensional cell patterning and is vital to the production of three-dimensional composite materials [58]. Tissue engineering is essential in the management of chronic skin disorders and burn injuries. An investigation employed the LAB technique to create fully cellularized skin, effectively utilizing various cell types in three-dimensional spatial configurations. Fibroblasts, keratinocytes, and Matriderm^®^ were employed to create skin substitutes, which were subsequently evaluated in vivo using nude mice. The data indicated that cells had experienced differentiation and proliferation, resulting in tissue creation, and notably, small blood veins developed towards the printed tissue from the wound bed and margins [59]. In an additional investigation, ADSCs were structured into a 3D grid pattern with LAB, and it was determined that cell proliferation and differentiation were unaffected post-printing. The expression of adipogenic markers indicated that the cell lineages resemble three-dimensional grafts comparable to real adipose tissue [60]. An investigation created injectable micro-scaffolds from electrospun material utilizing LAB that is capable of rapidly producing ten thousand micro-scaffolds with a high injectability rate. Furthermore, the cells were seeded into micro-scaffolds, revealing that these structures function as cell carriers and provide a more comprehensive investigation of minimally invasive cell therapies [61]. Even though the technology for this type of treatment is still in its early development stages, it has some promising potential and would also help alleviate existing organ-replacement transplantation shortages. Table 1 summarizes key bioprinting methods, focusing on their bioink compatibility, resolution, material deposition rates, and applications. The inclusion of metrics such as ‘Resolution’ and ‘Material Deposition Rate’ provides a clearer understanding of each technique’s capabilities and limitations in creating high-precision cancer models and other biomedical constructs. Table 2 highlights their unique advantages and drawbacks.

## 3. Opportunities Provided by 3D Bioprinted Cancer Models

### 3.1. Tumor Microenvironment Characteristics

**Figure 3:**
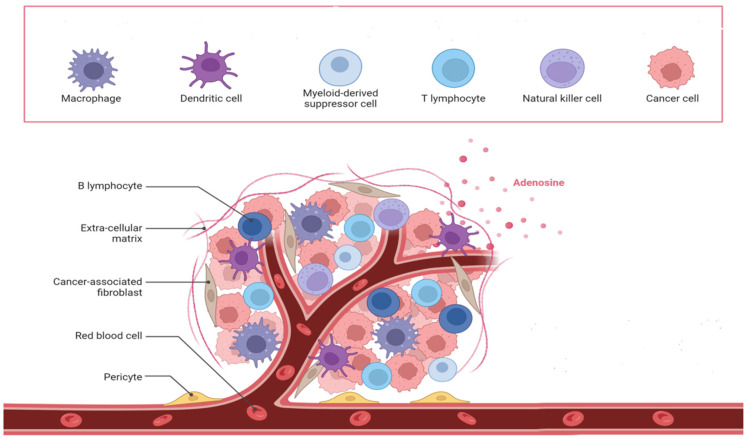
Characteristics of the tumor microenvironment. Tumors are defined by the presence of cells undergoing uncontrolled proliferation and are complex mini-organs comprising many cell types and matrix components. Created in https://BioRender.com.

Tumors are defined by uncontrolled cell proliferation and are complex mini-organs comprising several cell types and matrix proteins, as illustrated in Figure 2. Certain tumors may have almost 50% of their mass made up of nonmalignant cells [85,86]. Malignant cells actively engage with both elements of the tumor microenvironment [87]. In numerous cases, the accompanying nonmalignant cells facilitate cancer development via the production of substances such as cytokines and matrix remodeling enzymes. Lymphatic and immune system cells, blood vessels, pericytes, stromal cells, and adipocytes all contribute to the microenvironment, with their functions being consistent across many cancer types [88]. In the tumor microenvironment, populations of T cells, such as cytotoxic CD8+ memory T cells, may be located at the peripheries of infiltrating tumors and within the lymphatic drainage pathways of organs. Furthermore, CD8+ T lymphocytes that produce interleukin-2 and interferon-gamma (IFN-γ) are also present and frequently contribute to disease prognosis. Conversely, immunosuppressive T regulatory cells, such as CD4+ T cells that secrete TGF-β and IL-10, facilitate tumor development [89,90]. B cells also appear at the margins of tumors and in lymphatic structures, although both tumor-suppressive and tumor-promoting populations have been identified [91,92]. Natural killer cells are known to infiltrate the tumor stroma and may exhibit altered functions as a result of their interactions with tumor cells. Tumor-associated macrophages are a primary infiltrate in the tumor microenvironment and primarily exhibit tumor- and metastasis-promoting behaviors through the secretion of elevated amounts of IL-10. Macrophages possess a diverse array of angiogenic substances, facilitate neoangiogenesis in tumors, and engage in reciprocal contact with the surroundings. Tumors exhibit increased hypoxia, facilitating the recruitment of tumor-associated macrophages. The roles of several immune system cells, including myeloid-derived suppressor cells, tumor-associated neutrophils, and dysfunctional dendritic cells, inside the tumor microenvironment are being examined [86,93]. The other significant cellular alteration in the tumor microenvironment is the transformation of surrounding fibroblasts into myofibroblasts, facilitated through paracrine signaling pathways. Myofibroblasts contribute to cancer progression by inducing organ fibrosis. CAFs release mitogenic fibroblast growth factor (FGF) and insulin-like growth factor 1 (IGF1), while the epithelial−mesenchymal transition (EMT) stimulates TGF-β production. These fibroblasts additionally produce enzymes that facilitate ECM remodeling. CAFs may be organized in a core-branching configuration or may ring the tumor mass, frequently influencing the capacity of pharmaceuticals to access targeted malignant cells [94,95]. Vascular endothelial cells are another crucial cell type associated with the tumor microenvironment. The tumor vasculature significantly contrasts with normal vasculature in both structure and function characterized by disordered bifurcations, diverse lumens, and heightened permeability. The leakiness induces abnormal interstitial pressure and exacerbates hypoxia, ultimately promoting metastasis. Pericytes are frequently detected in many tumor microenvironments, providing structural support to the vasculature [96]. The extracellular matrix (ECM), in conjunction with the cellular component, is crucial in determining cellular fates within the tumor microenvironment [97]. ECM engages in dynamic interaction with the tumor cells to regulate the initiation and metastasis of malignancies. Modulation of cell−ECM adhesions is necessary for the cells to establish the microenvironment. The extracellular matrix serves as a storehouse for various growth factors. The spatiotemporal delivery of growth factors from three-dimensional matrices can be adjusted by matrix development. Growth factors are often generated by recombinant technology in bacteria or cells and necessitate rigorous standardization using techniques such as SDS-PAGE and ELISA examinations. From a physical perspective, tumor matrices exhibit increased extracellular matrix deposition by cancer-associated fibroblasts, leading to a greater elastic modulus than adjacent areas. Collagen and elastin fibers generate more rigid fibrils as a result of cross-linking facilitated by lysyl oxidase and transglutaminase. Matrix metalloproteinases, cysteine proteases, and cathepsins are the enzymes that facilitate the remodeling of the extracellular matrix in the tumor microenvironment. Furthermore, the extracellular matrix (ECM) comprises a variety of biomaterials exhibiting distinct mechanical and compositional characteristics that vary across different body tissues [98]. The mechanical characteristics of the microenvironment significantly influence cancer cell migration through the activation of mechanotransduction pathways. The migration of tumor cells in dense or unstructured 3D matrices has been confirmed to be specifically correlated with cellular stiffness [99,100]. Similarly, U373-MG human glioma cells exhibited differential migration behavior when the matrix modulus was adjusted between 0.4 and 120 kPa [101]. Therefore, the aforementioned discussion underscores the necessity of employing suitable fabrication techniques to accurately represent the intricate structure and functionality of the tumor microenvironment (Figure 3).

#### Enhanced Tumor Microenvironment

The tumor microenvironment consists of fibroblasts, epithelial cells, stroma, blood vessels, immune cells, signaling chemicals from both tumor and normal cells, and extracellular matrix (ECM) [102]. Despite advancements in 3D co-culture and microfluidic technologies, numerous obstacles persist in the development of tumor microenvironments. Recently, 3D bioprinting has been recognized as an innovative technique for constructing intricate tissue models with many biological applications, and we examined the function of 3D printing in the tumor microenvironment. A study showed that 3D glioma stem cells displayed improved capabilities to form spheroids, produce tubule-like structures, secrete VEGFA, and efficiently transform into endothelial cells. Furthermore, it was discovered that a 3D bioprinted hydrogel scaffold facilitates the requisite tumor microenvironment for glioma cells and glioma stem cells (GSCs) [103]. Acoustic droplet 3D printing operates without a nozzle, hence eliminating the risk of clogging. It enhances cell viability and elevates the quantity of cancer-associated fibroblasts within the tumor microenvironment, resulting in functional native tissue or pathological models [104]. A study on tumors and fibroblasts for generating a tumor microenvironment utilized a 3D-printed plastic microfluidic device, which facilitates heterotypic co-culturing and supports phenotypic analysis and molecular assays. The acquired data were confirmed through a mouse xenograft model, revealing that the 3D in vitro technique enhances the comprehension of carcinogenesis and the related tumor microenvironment [105]. A 3D-bioprinted GelMA/PEGDA hybrid scaffold replicated the tumor microenvironment of human malignant melanoma cells and was deemed appropriate for the tumor cells’ proliferation and differentiation; furthermore, the tumor cells exhibited accelerated growth and demonstrated drug-resistant capabilities [106]. Alginate and gelatin bioprintable hydrogel, in conjunction with BC cells and fibroblasts, were printed to create a three-dimensional model of a tumor microenvironment. This strategy increased cell survival and promoted the formation of tumor spheroids that interact with cancer-associated fibroblasts, offering an alternate paradigm to animal tumor models and 2D cultures for studying cancer biology [107]. In a different study, gastric tissue-specific bioinks, cellulose nanoparticles, and gastric dECM were employed to develop a tissue-specific microenvironment. The research showed that the utilization of cellulose nanoparticles enhanced mechanical capabilities, thereby augmenting gastric cell aggressiveness, and it may serve as an appropriate model for elucidating gastric cancer biology [108]. The influence of synthetic β-tricalcium phosphate structures on the interaction between neuroblastoma tumor cells and stromal components was investigated, revealing that the tumor microenvironment was influenced by the stroma and supported the proliferation of neuroblastoma cells. Furthermore, cytokine and fibronectin synthesis were induced, and the data elucidated how the 3D microenvironment prompts tumor cells to adopt spheroid morphology, thus enhancing the comprehension of metastatic neuroblastoma [109]. Another study utilized 3D printing technology to replicate the MCF-7 cell growth microenvironment with Cs/Gel composite scaffolds. The proposed scaffold demonstrated significant mechanical capability, improved biocompatibility, and was an accurate platform for drug screening. The impact of Geniposide was evaluated in a 3D culture system, revealing that cell growth was suppressed while cell apoptosis increased, indicating the anticancer properties of Geniposide [110].

### 3.2. Personalized Medicine

**Figure 4:**
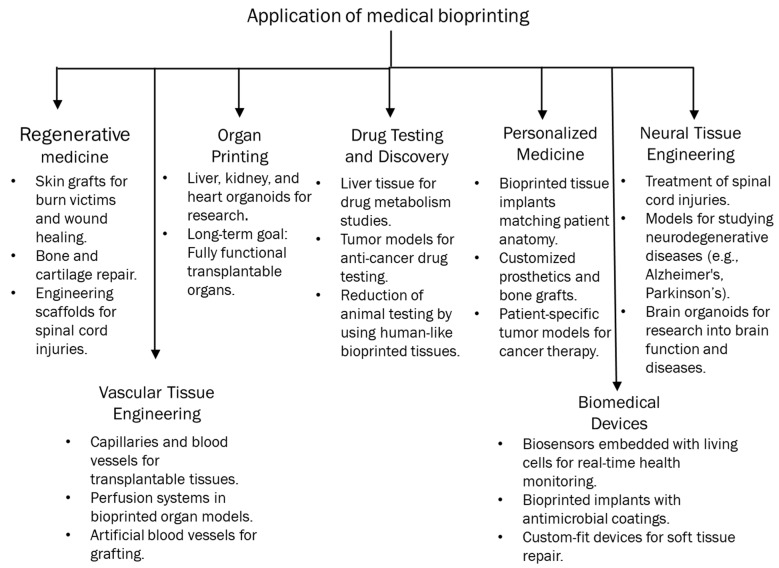
Classification and application of medical bioprinting.

Recent breakthroughs have contributed to the development of novel surgical instruments in cancer surgery. Nonetheless, achieving success with the requisite surgical techniques and perioperative management remains challenging. Recently, the application of 3D printing has facilitated surgical planning, minimized surgical duration, and enhanced therapeutic outcomes, underscoring the significance of 3D printing in cancer surgeries [111]. A study of 61 patients with right hemicolon cancer who had laparoscopic surgery classified them into three groups, control (n = 22), 3D printing (n = 20), and 3D-image (n = 19), to assess the significance of 3D printing in surgical procedures. It found that 3D printing significantly decreased surgical duration, blood loss, and the quantity of lymph node dissections, making it potentially more beneficial for beginner surgeons [112]. Patients who have undergone malignant pelvic bone cancer surgery (n = 12) via a 3D-printed bone-cutting guide and reconstruction with a 3D-printed implant were analyzed using clinical information. Three-dimensional printed guidelines facilitated patient recovery by yielding negative pathological results and promoting expedited rehabilitation. Furthermore, it was discovered that in contrast to the anatomical filling of bone deficiencies, 3D-printed implants may be fabricated and utilized [113]. Patients’ CT data were gathered for 3D reconstruction and printing, resulting in the development of 3D models to elucidate the relationship between a tumor and the hepatic bile duct, artery, portal vein, and hepatic vein, facilitating surgical planning and simulated procedures. The data indicated that liver failure or patient mortality was not identified perioperatively, eventually suggesting that 3D printing enhances surgical safety and mitigates surgical risk [114]. Three-dimensional printing was utilized on ten out of twenty patients who had endoscopic transsphenoidal surgery after being diagnosed with macroadenoma. The clinical findings indicated that patients utilizing 3D technology experienced shorter surgery durations, exhibited a lower incidence of complications, and ultimately benefited from an improved prognosis [115]. A separate study indicated that colorectal surgery might be enhanced by 3D printing technology, which improves patient education prior to stoma construction and aids in pre-operative surgical planning and assessment of liver metastases to chemotherapy via 3D ultrasonography [116]. Hong et al. [117] indicated that elucidating the thyroid gland, its anatomy, and surgical procedures poses significant challenges for doctors in their communication with patients. An investigation utilized a 3D-printed thyroid gland with cancer derived from CT scans of patients, which demonstrated the intricate structure of veins, arteries, nerves, and adjacent organs surrounding the cancerous growth. Consequently, this technology assists clinicians in educating patients and enhances comprehension of the disease. A hybrid 3D model of laparoscopic choledochal surgery was produced using a 3D Systems Project 660Pro with Visit PXL core powder. Nevertheless, the study indicated that additional refinement is required for this choledochal cyst excision simulation [118]. Three patients had their lung hilums 3D-printed, and sixteen patients’ pre-operative imaging were examined. The three-dimensional-printed hilum demonstrated superior accuracy compared to 3D-reconstructed CT, indicating that 3D printing is essential for thoracic surgical planning and offers greater advantages than traditional imaging techniques [119]. A research study investigated the efficacy of radioactive 125I seed (RIS) implantation, guided by CT and utilizing non-coplanar template 3D printing, in 66 patients with locally recurrent rectal cancer. The findings indicate that this method is a highly effective treatment strategy for patients post-surgery or external beam radiotherapy [120]. A randomized clinical experiment was performed to enhance patient understanding of informed consent for the utilization of individualized 3D-printed models in Stage I lung cancer surgery. According to assessments of patient knowledge, benefits, drawbacks, alternative therapies, and satisfaction, it was noted that personalized 3D printing may be applicable for patients with suspected stage I lung cancer [121]. Utilizing CT image source data for 3D printing is considered highly advantageous, as data obtained from CT machines cannot be printed without prior processing. A cost-efficient 3D printed skull was created, incorporating the nasal cavity’s shape at different stages of pituitary cancer, which can be utilized for surgical preparation of the endonasal trans-sphenoidal pituitary approach. Moreover, neurosurgeons and medical students can practice surgical techniques on various tumor stages [122]. A study was performed on lung cancer patients, categorizing them into several categories based on 3D chest CT reconstruction, 3D printing, and enhanced chest CT scans for 3D reconstruction. According to operation duration, bleeding loss, and post-operative problems, it has been discovered that comparative 3D printing techniques assist in accurately locating nodules and enhance surgical safety [123]. A 3D-printed model featuring a skull base, cerebral arteries, and a tumor/aneurysm was designed for a study during which 49 simulated surgeries were executed under a microscope, followed by an actual surgical procedure after acquiring expertise. The authors proposed that 3D-printed craniocerebral models adequately imitate surgical settings and assist in surgical planning, experience, and validation during actual procedures [124]. Figure 4 illustrates the categorization of medical bioprinting’s applications.

### 3.3. Drug Discovery and Screening

The development of cancer treatments is a formidable challenge, with just 5% of drugs successfully reaching the market [125] and an estimated cost of roughly USD 800 million [126]. This might be due to the inability of 2D cultures and animal models to replicate the in vivo tumor microenvironment, in contrast to 3D-printed cancer models [127], which also exhibit increased treatment resistance [128]. In recent years, there has been an increase in studies utilizing 3D printing technology for pharmaceutical development. Chen et al. created an innovative 3D-printed microfluidic device that can amalgamate several cancer therapeutics, potentially enhancing the efficacy of cancer treatment [129]. The aforementioned microfluidic chip demonstrates enhanced scalability, precision, and compactness, mostly due to the 3D printing capabilities to produce intricate and flexible designs [129]. Zhao et al. developed a 3D-printed model for anticancer drug screening utilizing gelatin, alginate, and fibrinogen as the matrix [130]. This study utilized hepatocyte and/or adipose-derived stem cells (ADSCs) to assess drug screening in 2D and 3D-printed models [130]. Multiple stains and three distinct medicines, namely 5-FU, astragalus polysaccharide (AP), and matrine, were employed in three groups with varying quantities [130]. Gelatin in different concentrations, both high and low, has been alternated within the matrix [130]. The investigation conducted by Zhao et al. indicates that the 3D model exhibits a stronger intercellular connection as cells migrate to the extracellular matrix, resembling the in vivo tumor microenvironment [130]. A low concentration of gelatin enhances cell–cell interactions in the 3D-printed model [130]. The concentration of anticancer drugs significantly influences cell survival and drug resistance; for example, 5-FU is more effective at low concentrations in reducing cell survival, whereas high concentrations result in a rebound effect. Additionally, the co-culture of hepatocytes and ADSCs demonstrates the highest level of drug resistance [130]. In comparison with 2D cell culture, a model developed using 3D printing technology is more likely to facilitate high-throughput, scalable, and dependable drug screening [130].

## 4. Challenges Facing 3D Bioprinted Cancer Models

### 4.1. Technical Challenges in 3D Bioprinting

Numerous technological challenges are linked to the application of 3D bioprinting for the creation of cancer models. 3D biofabrication technologies have consistently advanced to enhance microresolution printing. Exceptional anatomical detail is achieved using photolithographic manufacture employing laser scanning or digital light processing alongside two-photon polymerization, microfluidic chip-mediated techniques, and ionizing radiation [131]. Besides the quality of high-definition printing, the presence of numerous live biological cells poses an additional challenge. Following printing, it is crucial that the cells retain their functionality and vasculogenic properties throughout time. Vascular bioprinting poses a challenge in vascular perfusion. Three-dimensional bioprinting of materials facilitates luxury-level cancer cell behavior at the microscale. Organ or tissue systems comprise populations of capital goods at varying densities. Cellular aggregates must be vascularized in a 3D bioprint to provide robust blood and oxygen circulation. The application of 3D printing technology in intricate, multicellular systems, including organoids or pregnancy cultures, is crucial [132]. Moreover, bioprinting entails intrinsic variability, necessitating the establishment of operational frameworks and methodologies that ensure consistent variables. Troubleshooting room temperature presents several opportunities for bioreactor variation. Reproducibility is seldom addressed; nonetheless, a qualitative and functional bioprinting model, particularly one designed for scale-up, necessitates the use of polymers that are both robust and reproducible. To instill confidence in professionals regarding the authorization of new medication development processes or findings, it is essential to establish standardized technologies and methodologies. Advancement necessitates a thorough comprehension of the discourse and recommendations regarding the execution of bioprinting. The computational challenges are propelling cancer model technology towards more effective techniques [133].

### 4.2. Reproducibility of 3D Bioprinted Cancer Models

Reproducibility is fundamental to scientific inquiry. The capacity to provide consistent data across several biological investigations signifies the potential reliability of their results. A major difficulty currently confronting 3D bioprinted cancer models is their low repeatability. Establishing a reliable model that yields the same outcomes upon rejuvenation can be challenging. Consequently, investigations into diverse technical challenges pertinent to this research domain must incorporate this aspect and replicate the disease process or model to yield statistically accurate results. Overcoming this challenge necessitates the development of dependable and adaptable bioprinting methods. Biological materials have intrinsic variability; nevertheless, by selecting an appropriate combination of cells, hydrogels, and bioinks, one can effectively mitigate or manage this variability to an acceptable degree [134]. Variability may arise from various sources, including discrepancies in material properties, especially in printed gel configurations, which could stem from changes in crosslinking, environmental factors, or cellular viability. Variability can be minimized, if not entirely eradicated, from the printing process. The inherent unpredictability of the printing method and materials necessitates careful selection or design of printing parameters in the development of 3D bioprinted cancer models. It is essential to document and report the procedure with adequate detail to ensure its reproducibility. This will mitigate unpredictability resulting from technical discrepancies in print configuration. Adhering to a standard operating procedure enables the regulation of these bioinks, facilitating reproducibility among various bioinks as a measure of quality control. Moreover, the printing technology must effectively handle ergonomic concerns to integrate with standard cell culture infrastructure. This necessitates the operator’s perspective. The design of models within the realms of tissue engineering and materials science necessitates a perspective akin to a ‘beauty contest’: there is no definitive ‘winner’, yet as long as there is a consensus on the criteria for success, collective advancement is achievable. In constructing a 3D bioengineered cancer model, it is imperative to clearly define our objectives. We can proceed to devise optimal methods for creating a replicable model. Consequently, the inquiry needs to be, ‘The reproducibility of what?’ The veracity of this response will propel the discipline forward. Researchers must reach consensus on the protocols for model development, hence augmenting the practical use of the discipline. Certain domains of 3D cell culture are expected to gain more from enhanced inter- and intra-laboratory reliability than others. Entities that directly influence disease control are likely to derive the greatest advantage, although this has to be determined. The previously identified application areas provide insight into the current priorities in the field. Consolidating a standardized document will enhance reproducibility for individuals unfamiliar with biological work and facilitate the usage of 3D printing in their scientific endeavors. This will significantly strengthen the utility of 3D printing and bioprinting techniques within the broader domain of cell culture. The enhanced reliability among groups will facilitate the unequivocal clinical translation of research. Partnerships between printing firms and research institutions may yield potential alternatives; however, it is regrettably improbable that this will occur in the foreseeable future [135].

### 4.3. Standardization of Protocols

Despite significant advancements in the development of complex biological models, an attrition rate of over 90% persists between preclinical and clinical trials. A significant obstacle to advancement is the absence of standardized techniques for the development of 3D bioprinted cancer models. The various protocols employed for materials, printer configurations, or post-processing techniques in the fabrication of 3D cellular structures can significantly influence the uniformity and ultimate biological outcomes of these models. Consequently, there is a need for more comprehensive and reproducible methodologies for model creation to ensure uniform and consistent outcomes from both independent laboratories and multi-entry studies. Standardization and the establishment of industry-wide standards for materials and techniques could enhance efficiency, facilitate cancer modeling, replace animal trials, and yield disease-relevant and physiologically meaningful outcomes [136,137,138]. 3D bioprinted cancer models are becoming acknowledged for their significant differences in characteristics and results relative to 2D cultures, especially in drug reactivity testing. The fabrication processes used to create these bioengineered models may provide challenges for their transition from fundamental research to practical use. Diverse workgroups and researchers may leverage their distinct knowledge, with certain techniques trademarked and hence inaccessible to the community. Consequently, study results may vary significantly between laboratories, rendering comparisons challenging and models impossible to employ effectively. Establishing a consistent methodology for printers, software, and materials will enable researchers to swiftly transition from a recognized platform to assess their alterations, hence enhancing productivity and efficiency [139,140].

### 4.4. Bioink Limitations

Despite considerable advancements in the development of bioinks, obstacles and limits persist in the fabrication of optimal hydrogel-based bioinks for cancer simulation. Currently, hydrogel-based bioinks are inadequate for accurately mimicking the tumor microenvironment (TME) in vitro due to trade-offs in bioprinter compatibility, biocompatibility, slow gelation dynamics, shear thinning properties, and suboptimal printing resolution, hindering the effective preclinical development of cancer therapies. TMEs are intricate and dynamic, consisting of numerous components that are organized geographically and temporally, which collectively influence tumor growth, invasion, and metastasis. Nevertheless, hydrogel bioinks remain significantly inadequate in replicating natural tumor microenvironments. The hydrogels employed for bioprinting cancer models must exhibit specific characteristics. Structural stability and print resolution of bioinks are critical considerations in the design of bioinks for cancer model printing. The matrices must possess an extended gelation time to facilitate the 3D–2D migration of multi-cancer cell populations post-printing, while simultaneously achieving shear thinning to enhance cell handling and rapid gelation after printing, thereby preserving the printed shape for subsequent cell phenotypic performance analysis. Thus, it is crucial to combine excellent printability with the preservation of the intended shape. The trade-off in bioink development presents a limitation for the advantageous properties of hydrogels in bioprinting. Moreover, for multi-material printing requiring the development of bioinks compatible with bioprinters, multiple printability assessment methodologies should be taken into account in bioink innovation [141,142,143].

#### 4.4.1. Biocompatibility Issues

Bioinks utilized in 3D bioprinting must be compatible with cells and facilitate an environment conducive to cell growth, proliferation, and differentiation. Certain applications may necessitate support functions such as conduction or medication release. Various applications necessitate distinct specifications for the composition, structure, and characteristics of bioinks. Researchers have performed comprehensive studies, and typically, there are bioinks tailored for certain applications. Biological tissues typically comprise a multitude of diverse cells and their corresponding extracellular matrix. These bioinks are utilized to create 3D structures via 3D printing technology, emulating the natural tissue of the human body. The biological bioink typically comprises cells and the extracellular matrix. The classification of cell types is based on the distribution of cell composition within the tissue or organ designated for repair. At present, stem cells are the preferred option. The optimal biological bioink serves as an effective carrier for cells, offers an appropriate environment for cellular proliferation, and closely mimics the natural tissues of the human body [144,145,146]. Nevertheless, the specifications for bioink frequently conflict in practical usage. To ensure that the printed cells preserve their biological activity and reproduction rate, the extracellular matrix network within the bioink must exhibit excellent biocompatibility. To ensure the bioink remains continuous during 3D printing and is capable of producing intricate structures of varying shapes, its physical qualities must be exceptional. Simultaneously accomplishing both is a significant challenge. Consequently, mechanical stability, the presence of a chemical crosslinking agent, and certain synergistic effects, along with the interaction between the cell and the 3D printing system, as well as the adjustment of bioink parameters throughout the 3D printing process, frequently result in diminished biocompatibility. In recent years, researchers have attained specific outcomes in artificially synthesized materials inside the sector, yet these materials require enhancement when juxtaposed with biological natural materials. Artificial materials frequently exhibit inferior biocompatibility compared to natural materials. In comparison to biodegradability, synthetic materials are significantly inferior. Biological tissues excel in both domains [147,148,149].

#### 4.4.2. Mechanical Properties

Bioinks for bioprinting must have adequate rheological and mechanical qualities to guarantee proper gelation throughout the printing process. Furthermore, the printed biocomposite must maintain high shape accuracy and resolution to accurately replicate intricate biological interactions. This encompasses the dimensions and hierarchical arrangement of cells and their extracellular matrix, particularly the rigidity of the supportive structures created. Bioinks exhibiting plastic and elastic deformation properties suitable for cellular structures while preserving dimensional stability are essential in this context. In cancer cell biology research, cells must be printed within a soft matrix that accurately replicates the tissue-level mechanics of the underlying tumor environment. Mechanical qualities are crucial for cellular preservation, encompassing adhesion, survival, migration, and participation in specialized tasks [150,151]. Tumor tissue exhibits different degrees of stiffness, with the periphery becoming rigid while the middle region softens in in vitro two-dimensional monolayer cultures or conventional three-dimensional bulk investigations, rendering specific interactions incomparable. To preserve the whole range of cellular behaviors, including survival, motility, and responses to chemokines implicated in malignancy, it is essential to sustain a soft tissue-like microenvironment for cancer cells. It is crucial to acknowledge that certain tumor cell types tend to selectively infiltrate stiffer surroundings akin to the rigid fibrotic milieu of a primary or metastatic tumor. The development of hydrogels tailored to mimic differences in tissue-level mechanics is essential for the printed cancer model. Furthermore, the physiological environment must be engineered to replicate in vivo conditions, ensuring adequate breathability, sufficient transparency, and the capacity to facilitate oxygen and nutrient circulation, as well as efficient waste elimination [152,153].

## 5. Current Advances and Emerging Solutions

### 5.1. AI Optimization of Bioprinting

**Figure 5:**
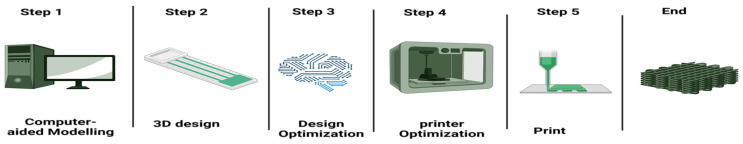
The 3D printing process demonstrating the steps that require optimization protocols (Designed with Biorender).

Three-dimensional printing needs to be optimized for enhanced functionality, reduced costs, and decreased energy consumption. Simultaneously, optimization is an essential process for a more precise evaluation of the material attributes and specifications required. Regarding the application, in the optimization of 3D printing, it is essential to consider variables such as the material’s structural properties, the object’s volume, shape, and dimensions, temperature fluctuations during printing, pressure, and environmental conditions [154]. Machine learning (ML) represents an innovative paradigm in the optimization of bioprinting and constitutes a subset of artificial intelligence (AI). Unlike conventional computational optimization, machine learning algorithms aim to execute “intelligent” tasks by discerning patterns within data; conversely, most conventional methods depend on explicitly constructed models [155] (Figure 5).

Process optimization is typically conducted prior to printing. The procedure can be predicted using machine learning. Information on process optimization can be acquired by inputting the properties of the material, including density, melting temperature, and freezing temperature, as well as the characteristics of the object to be printed, such as volume, thickness, length, and shape. Factors including needle tip size, printing speed, pressure, printing temperature, cost, and duration can be assessed prior to printing. Consequently, these estimations yield more economical and higher-quality 3D prints with reduced deformation [156]. The identical outcomes pertain to 3D bioprinting. In the 3D cell bioprinting process, machine learning facilitates the prediction of parameters such as cellular damage and cell density per area. Moreover, researchers and algorithm developers can collaboratively enhance existing algorithms utilizing the cloud system. Thus, process optimization can yield increasingly stable and precise results over time. Typically, four methodologies of machine learning may be employed for optimization. These are referred to as supervised learning, unsupervised learning, semi-supervised learning, and reinforcement learning. Moreover, a deep learning approach may also be employed.

The first design phase of 3D bioprinting typically includes acquiring images from patients to inform the construction of the 3D print [157]. Raw imaging data of specific tissues or organs is acquired using medical imaging methods such as magnetic resonance imaging (MRI) and computed tomography (CT), which provide sequential images of the targeted areas. The next phase is the precise segmentation of these pictures, in which specific regions of interest inside the organ are identified and separated from the surrounding tissue. The segmented data are subsequently imported into computer-aided design (CAD) software, enabling the reconstruction of a digital 3D model of the organ. This model functions as a framework for generating print paths and printing parameters for the bioprinting process [158,159,160]. The precision of each stage is essential to the quality and operation of the printed structures. Conventional medical image segmentation and processing techniques are frequently laborious and susceptible to errors [158]. Artificial intelligence has implemented automatic segmentation algorithms and augmentation tools that substantially decrease manual input, enhance efficiency, and eliminate errors. Traditional AI has been employed to augment and automate the operations of image enhancement, segmentation, medical picture reconstruction, and print path development [161,162,163]. Bouzon, Albertini, Viana, Medeiros, and Rodrigues [164] utilized a bio-inspired algorithm in conjunction with computer vision and graphics techniques for the reconstruction of medical images, which poses significant challenges in unstructured environments like inside human organs. The methods improved picture segmentation by decreasing entropy, identifying critical points, and eliminating mismatched point pairs, thus enhancing the quality of reconstruction. Sainz-DeMena, García-Aznar, Pérez, and Borau [161] have created the im2mesh Python (ABAQUS (Dassault Systèmes, Paris, France) or ANSYS (Ansys, Inc., Pittsburgh, PA, USA) library to automate the conversion of segmented slices into intricate 3D meshes, incorporating scattered data via slice interpolation and enhancing the usability and integration of these models into patient-specific simulations. The resulting 3D mesh exhibited superior intersection over union (IoU) scores when compared to surfaces produced by established applications such as 3D Slicer. The reconstructed 3D model has enabled classic AI to automatically produce a viable print path. Nguyen, Phung, and Bui [165] used computer-aided process planning systems with macro programming techniques to automate and improve the creation of G-codes for CNC machining.

This technology has improved the flexibility, customizability, and speed of the process through the identification of the geometric characteristics of the CAD model and the subsequent creation of G-code. Machine learning approaches, proficient in pattern identification and predictive analytics, have been employed to optimize picture segmentation in medical image reconstruction. Roth, Oda, Zhou, Shimizu, Yang, Hayashi, Oda, Fujiwara, Misawa, and Mori [166] have created cascaded fully convolutional networks for the segmentation of CT images. It employs a two-stage, coarse-to-fine methodology that facilitates the successful segmentation of intricate anatomical structures across diverse scales, ranging from enormous organs to slender capillaries. This method enhances segmentation, especially around the edges of tiny organs and vessels, and dramatically increases the mean Dice score, indicating a marked improvement in segmentation accuracy. Chowa, Azam, Montaha, Bhuiyan, and Jonkman [167] presented an innovative technique that converts conventional 2D ultrasound images into 3D meshes, effectively preserving intricate geometrical characteristics of breast tumors frequently overlooked in standard imaging methods by extracting clinically pertinent features from these 3D models and employing a graph attention network.

**Figure 6:**
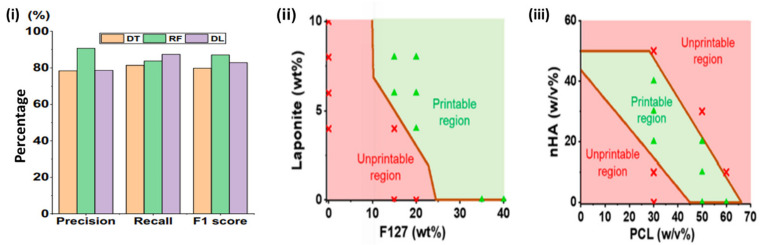
The application of machine learning to material selection for bioprinting. Machine learning for forecasting the printability of inks comprising hydrogel-based and polymer/organic solvent systems. (**i**) Evaluation measures including accuracy, precision, recall, and F1 score for machine learning models in predicting printability. (**ii**) The anticipated printability table of the F127/Laponite hydrogel nanocomposite. (**iii**) Polycaprolactone polymer nanocomposite ink filled with hydroxyapatite nanoparticles. Reprinted from ref. [171].

**Figure 7:**
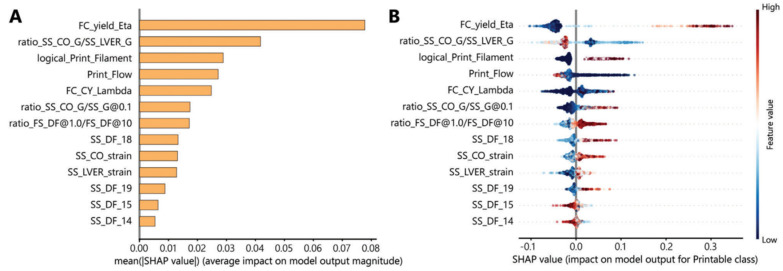
SHAP values for various aspects (rheological properties) of inks evaluate their contributions to predictions on a global scale (**A**) and a local scale (**B**). A greater value signifies a more substantial contribution to the alteration in printability. Reprinted from ref. [172].

Machine learning offers an innovative methodology for ink development by analyzing extensive information and identifying patterns that may be deemed too intricate for human comprehension [168,169]. The printability of inks is a crucial characteristic of 3D printing, as it significantly affects the integration and functionality of produced implants [170]. The evaluation is conducted by examining the shape fidelity of printed objects. Machine learning has been employed to predict the printability of biomaterial compositions, facilitating the advancement of bioinks. Chen, Liu, Balabani, Hirayama, and Huang [171] have employed machine learning methods to forecast printable biomaterial formulations for direct ink writing (DIW), an extrusion-based printing method. The study’s data include 210 ink formulations utilizing two ink systems (hydrogel-based and polymer organic solution-based), 16 biomaterials, both natural and synthetic, and four solvents. The biomaterials comprise polymers of various molecular weights and characteristics, as well as functional fillers of diverse sizes and functionalities. The inks were 3D printed into four-layer lattice structures via the direct ink writing (DIW) technique, and their printability was evaluated. The machine learning algorithms, including decision trees, random forests, and deep learning, have effectively predicted the printability of biomaterial formulations with high accuracy (>88%), as illustrated in (Figure 6). A printability map of biomaterial composites can be produced using the learned machine learning algorithms to inform the ink design (Figure 7A). This study has facilitated the application of machine learning in the selection of materials with diverse properties for direct ink writing 3D printing, including hydrogels for bioprinting. Nadernezhad and Groll [172] employed a random forest method to forecast the printability of hyaluronic acid-based hydrogel inks based on their rheological properties. The significance of various rheological features was quantitatively evaluated, leading to the identification of 13 essential rheological measurements that determine the printability of hydrogel formulations (Figure 7B). Statistically, the trained model forecasts that a printable formulation must demonstrate elevated yield viscosity and reduced plasticity prior to flow. The transition from Newtonian to non-Newtonian flow properties should occur at relatively modest shear rates.

The existing bioprinting method entails the manufacture of a cellular tissue structure at the production site, which is subsequently transported to the surgical room for transplantation [173,174]. Despite its significant potential, inherent limitations of traditional 3D bioprinting may impede the realization of its full capabilities. The bioprinting process usually occurs on a flat substrate; nevertheless, the defect site is frequently uneven and complex, resulting in challenges in attaining geometric alignment between the printed and target surfaces. The processes of handling, transporting, and implanting present hazards of harming the micro- or macro-architectures of the mechanically fragile bioprinted cellular structures. The potential for contamination during these processes is a significant concern [174,175,176]. In situ 3D bioprinting has arisen to tackle these issues. This entails the direct application of bioinks to the defect site within a therapeutic environment for the regeneration of tissues or organs by a minimally invasive approach. This approach can regenerate and rebuild damaged tissues with non-planar surfaces or intricate geometries. Following printing, the human body functions as an “in vivo bioreactor,” facilitating the post-printing development and maturation, hence obviating the necessity for an artificial milieu for the maturation of bioprinted items in vitro using bioreactors [174,177]. Surface acquisition and print route planning are essential for in situ printing. Accurate assessment of surface geometry guarantees precise material deposition, while effective print path planning optimizes the printing process for speed, material efficiency, and product quality. Classical AI systems have been employed to obtain geometric data, generate printing trajectories, and integrate intelligent printing mechanisms.

**Figure 8:**
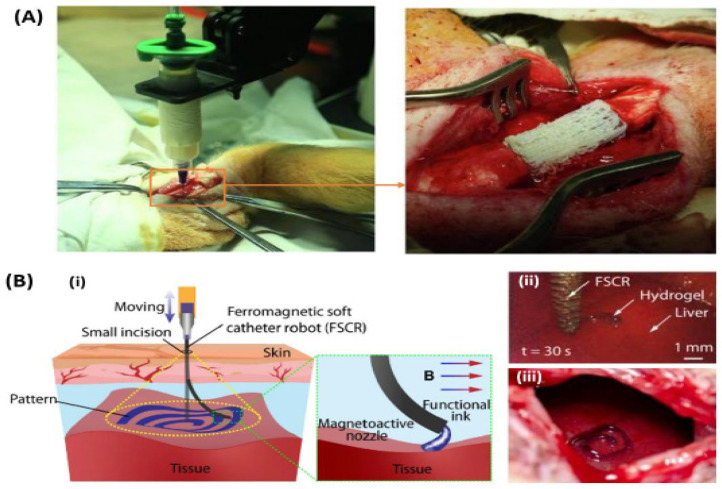
AI-guided in situ 3D bioprinting with open-loop system. (**A**) In situ bioprinting on a porcine long segmental bone defect using a robotic manipulator-based 3D printer. A porous bone scaffold was printed in the bone defect. Reprinted from ref. [181]. (**B**) Schematics illustrate the minimally invasive printing process using a ferromagnetic soft catheter robot (FSCR) system. Functional inks, such as conducting polymers and living materials, are printed through the skin via a small incision. (i) Minimally invasive printing of conductive hydrogel on the liver surface (**B**) represents the magnetic field and (ii) resulting printed spiral pattern. (iii) Reprinted from ref. [182].

This has facilitated in situ three-dimensional printing on intricate and non-planar surfaces [176]. Zhu, Ng, Park, and McAlpine divided these AI systems into open-loop, closed-loop, and predictive systems in their review study. In open-loop AI systems, the target surface remains static, and the geometry is predetermined to create the print path. In a closed-loop AI system, a feedback-control mechanism adjusts the print trajectory during the printing process, enabling 3D printing on dynamic surfaces. Figure 3 illustrates a schematic depicting the application of AI in 3D bioprinting. Open-loop 3D printing entails acquiring data regarding the target surface geometry prior to the offline printing process. Consequently, AI can evaluate this geometric data to formulate the print route and ink deposition for in situ printing. Diverse imaging modalities, including CT scanners [178], laser scanners [179], and structured-light scanners [180], have been employed to get the three-dimensional geometry data of the target surface. Li, Shi, Ma, Jin, Wang, Liang, Cao, Wang, and Jiang [181] employed a robotic manipulator-based 3D printer for in situ printing within living animal models (Figure 8A). They conducted 3D scanning to obtain the extensive segmental defect surface on the tibia of pigs for path design through reverse engineering. Porous scaffold constructions were fabricated on the faults within 12 minutes (Figure 8(Bi)). Micro-CT scans demonstrated that the 3D bioprinting group presented a continuous cortical bone structure at the defect site after three months, while the control group exhibited gaps and voids. Histological investigation revealed enhanced morphology of the regenerated bone tissue in the 3D bioprinting cohort. This illustrates the viability of using the robotic printer to mend extensive segmental bone lesions. Zhou, Yang, Wang, Wu, Gu, Zhou, Liu, Yang, Tang, Ling, Wang, and Zang [182] developed a ferromagnetic soft catheter robot (FSCR) system for intelligent and minimally invasive bioprinting (Figure 8(Bii)). The FSCR employs magnetic actuation and is capable of remote control, facilitating accurate and automated printing of diverse materials through a little incision. The three-dimensional surface of a living rat’s liver was recreated using computed tomography, and a printing trajectory was delineated on the superior surface of the liver. The FSCR effectively deposited conductive hydrogel in a spiral configuration on the liver’s surface in vivo within 70 seconds (Figure 8(Biii)).

**Figure 9:**
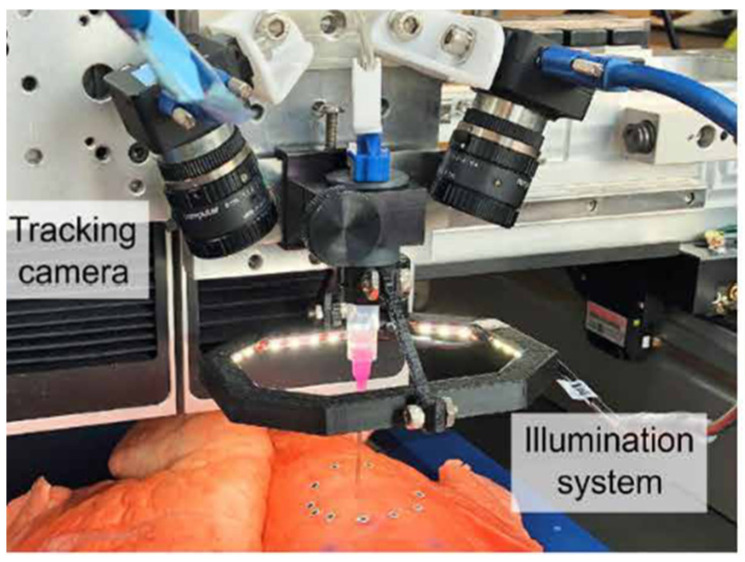
AI-assisted in situ three-dimensional bioprinting using a closed-loop system. Three-dimensional printing of the conductive hydrogel layer on a lung model experiencing respiratory motion. Reprinted from ref. [184].

The closed-loop AI methodology for in situ printing entails online 3D printing with real-time adjustments to operational variations such as target surface movement and deformation, printing errors, ink flow irregularities, and nozzle performance [176]. This facilitates enhanced printing quality via real-time adjustments and on-site printing on dynamic surfaces. Zhao, Hu, Lin, Wang, Liu, Wang, Zhu, and Xu [183] devised a closed-loop, minimally invasive in situ bioprinting methodology. The system comprises a 7-axis bioprinting robot, a binary chromatic ring array (BCRA) marker for trocar identification, a vision system for monitoring trocar movement, and a real-time control component. The BCRA marker guarantees accurate placement and assessment of the trocar, while the bioprinting robot directs the end-effector through the trocar to perform procedures within the small incision. The vision system records the trocar’s orientation, which is subsequently utilized for real-time alignment and correction of the printing end-effector’s orientation. The method has fabricated hydrogels via an artificial skin on a porcine liver model with submillimeter precision, ensuring low contact force during incisions and enhanced adaptability for intracorporeal procedures influenced by respiration. Zhu, Park, and McAlpine [184] have created a system that can estimate the real-time motion of the target surface and adjust the printing process accordingly, enabling in situ 3D printing on a dynamic lung model (Figure 9). A stereo camera system was employed to monitor fiducial markers on the tissue surface within a real-time shape-based model to assess the deformation of the target surface. The system may dynamically adjust the printing process based on the real-time geometry conditions of the substrates. They effectively printed conductive hydrogel onto a pig lung and incorporated it into a strain sensor capable of continuous spatial tracking of deformation. This adaptive 3D printing method has the potential to improve robot-assisted medical therapies and facilitate the direct printing of bioinks on and within the human body for tissue regeneration or wearable electronics applications.

### 5.2. Hybrid Techniques in Bioprinting

Using a standard bioprinter based on a macroscopic 3D bioprinting technology in conjunction with a microfluidic device, hybrid bioprinting enables highly precise spatial assembly of cells and gel growth factors. The scaffolds’ biofabricated heterotypic tissue zones, nutritional upkeep, and metabolite waste elimination can all be supported by an embedded microfluidic network. The benefits of both bioprinting methods are combined in hybrid bioprinting. Combining bioprinting and microfluidic technologies, microfluidic bioprinting enables drug screening, miniature tissue models, and the highly accurate dispensing of biochemical components. For drug screening, organ reproduction, and single-cell resolution analysis in regenerative medicine, 3D tissues, organoids, and functional cell–matrix interactions can also be bioprinted in situ. Successfully filling delayed gelation hydrogels or being severely impacted by shear stress and obstruction are issues in three-dimensional bioprinting systems [185]. It is possible to alter the pH of the helping hydrogel and generate the ionized amino gel for the bone-like structure without raising the temperature by adding the proper mineral acid. Sodium alginate, carrageenan, and pre-made mussel-inspired micro-silica are combined to create a possible precursor ink for direct extrusion bioprinting. Channel-cable, multi-level, multi-material, and gradient material assembly are features of hybrid-scale 3D bioprinting scaffolds. By carefully regulating the cell placement at the micro-scale resolution and printing gel scaffold structures, modern 3D bioprinters can print many cell types immersed in their remarkable hydrogel in multi-layer patterns. The gel network can maintain cell growth to accomplish a multi-level assembly and adapt to the 3D cell culture landscape in vivo after undergoing bio-maturation in the incubator. The printing format results are mostly restricted to the production of inorganic ceramics, but the system can process colloidal inks and pastes and offers spatial diversity in the body design and granularity. The printing of several components at the microscale while creating realistic chemical and physical microenvironments in three dimensions remains a considerable problem, despite the development of numerous bioprinting methods. With the ability to print live cells, biodegradable biomaterials, and cell–matrix-stimulus multi-components at high resolution in living subjects, in situ bioprinting can be regarded as a novel tissue engineering technique that produces intricate, biocompatible, and tissue-mimetic three-dimensional forms. This multi-stage technique can produce UV-started gels, such as those with high humidity and biocompatibility, as well as thermally initiated gels with cross-linking agents and chemically catalytic curable gels. Cells made of thermopolymerizable materials can form in situ, multi-layered, three-dimensional tissue nanostructures in a variety of sizes by encasing growth factors and support structures in multilayers. Thus, by integrating microfluidic principles that may create a wide variety of shapes, the aforesaid item improves the ability to include cells [186,187,188,189].

### 5.3. Microfluidics in Bioprinting

Microfluidics pertains to systems that manage small volumes of liquids, possess a high surface area for confined flow, and exhibit flow characteristics predominantly defined by laminar flow. Diverse bioprinting matrices and cell types have been employed in microfluidics, with numerous innovative approaches and foundational studies documented. One facet of the study is the production of biomaterials for microfluidics bioprinters, with the objective of producing high-resolution, rapid-response, and stimuli-responsive substrates. A singular example can illustrate the research methodology in biomaterials development. Viscoelastic droplets have been employed to illustrate gas sensing on a flexible polymer substrate, and the outcomes of UV irradiation exposure confirm that the viscoelastic droplet remains entirely intact without degradation [190,191]. Continuous attempts are underway to develop novel bioink compositions to enhance cell viability. A novel polymer is developed to offer distinct attributes, including enhanced resolution, superior mechanical strength, and great transparency. In certain microfluidic methodologies, matrices have been developed to enhance cell spreading and optimize cell–matrix interactions by regulating cellular alignment and other characteristics. Cells can exert pressure to elongate their hanging nuclei. Numerous cellular processes are intimately linked to cell spreading; hence, elongated cells can facilitate biosensing of their environment. The stretching behavior of preosteoblasts is demonstrated in their findings [192,193].

### 5.4. Advanced Formulations of Bioinks

Bioinks can be formulated using several polymers, ceramics, and non-matriomorphic substances. Polymer-based bioinks are extensively utilized owing to their straightforward composition, manipulation, and market accessibility. These are frequently hydrogels originating from natural polymers, including gelatin, collagen, hyaluronic acid, alginate, fibrin, or photo-insensitive synthetic polymers such as polyethylene glycol, poly(lactic-co-glycolic acid), or polyacrylate. Ceramic-based bioinks, consisting of hydrogels infused with inorganic nanoparticles, frequently incorporating hydroxyapatite, exhibit potential for application in bone models. Non-matriomorphic bioinks consist of cells encapsulated in carrier droplets, which are printed in conjunction with biocompatible inks that facilitate cell seeding, proliferation, self-assembly, and colonization. While polymer-based bioinks are frequently advantageous for cellular proliferation, their synthetic composition presents a disadvantage by compromising the interaction between the hydrogel and cells, obstructing cell motility, constraining biological responses, and perhaps influencing the intended cellular phenotype [194,195]. Notably, synthetic polymers typically inadequately replicate the natural extracellular matrix compared to naturally generated hydrogels, rendering them less favorable for cellular functions. To address these limitations, extensive research has focused on creating innovative biomaterials, resulting in the development of numerous advanced bioinks. These can be tailored in various domains, including physical, chemical, and biological characteristics, as well as functionality, utilizing advanced assisting technologies such as micropatterning, cross-linking, co-printing, or electrospinning, and/or directly integrating biologically functional molecules before or after printing. Furthermore, prevascularization studies have propelled the creation of composite bioinks, which consist of tissue-specific cell-laden hydrogels and sodium alginate pericellular capsules containing releasing factors. Benefits have been observed from the improved vascular supply of transplanted 3D bioprinted tissues. As a result, recent advancements in bioinks have shown diverse stimuli-responsive characteristics, customized structural designs, and distinctive biofunctionality, providing promise for tissue organ regeneration [196,197,198].

**Figure 10:**
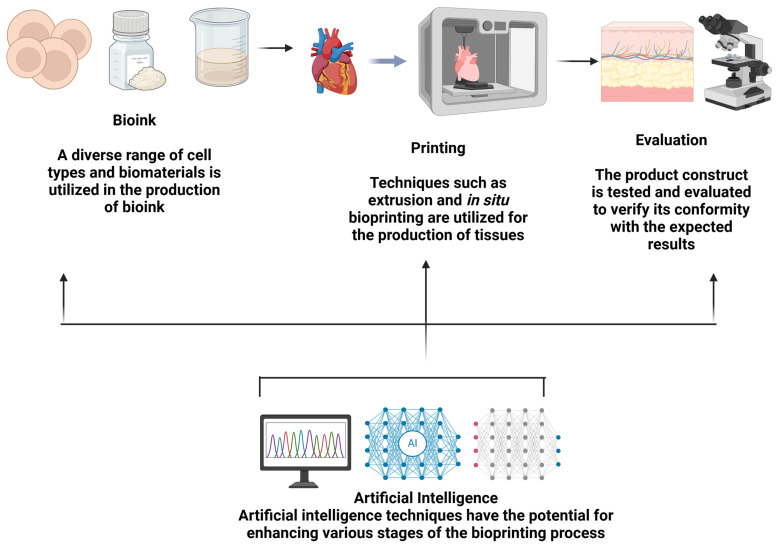
Three-dimensional bioprinting: current approaches, technological advancements, and the role of artificial intelligence. Created in https://BioRender.com.

This topic can be summarized in Figure 10.

## 6. Future Prospects and Implications for Cancer Research

Despite the significant potential of 3D bioprinting in the efficient and economical production of intricate in vitro tumors, numerous hurdles persist in advancing this technology within personalized oncology. Currently, existing 3D bioprinting techniques are unable to accurately replicate the intricate architecture of the numerous tissues within the human body. Moreover, the availability of biomaterials for 3D bioprinting is restricted, frequently lacking the ability to accurately replicate the characteristics of genuine tissue. To effectively integrate additional natural biomaterials into the bioprinting process, collaboration among chemists, materials engineers, and other professionals is essential. Moreover, technical obstacles pertaining to printing resolution and speed must be surmounted. Regulatory agencies are encountering difficulties in converting tumor models for personalized or precision medicine, which threatens the timely integration of bioprinted items into clinical practice, similar to the challenges faced with animal models and other reduced models. Biofabrication is inherently a multidisciplinary field, as is the advancement and enhancement of novel technologies for customized oncology. Interdisciplinary collaboration is essential to overcome existing restrictions and advance 3D bioprinting in oncology. Collaboration across multidisciplinary research teams can enhance the consistency and predictive validity of 3D bioprinted tumor models. Engineers and chemists are urged to integrate various biofabrication techniques to substantially improve the tumor model and ultimately establish a novel paradigm in cancer treatment. Indeed, distinct limitations emerge, particularly regarding cellular compartmentalization and the imperative to use microfluidics inside integrative body-on-chip investigations. Moreover, 3D bioprinting techniques involving post-printing or post-differentiation stages that necessitate additional tissue maturation are exceptionally efficient as they provide enhanced regulation of the intricate arrangement of various cell layers or vascular structures. An instance of the latter technology is the integration of photoreactive extrusion-based bioprinting. Significant advancements pertain to the incorporation of artificial intelligence to enhance the precise deposition of cells and distinctive microphysiological bioprinted systems [31,199,200].

## 7. Conclusions

Three-dimensional printing technology, in its advanced application within oncological research, facilitates not merely the replication of tumors’ three-dimensional spatial configuration but also the emulation of the tumor microenvironment, thereby revealing cancer’s intrinsic attributes. This dual capability significantly enhances the exploration of cancer’s etiology and progression, fosters the translation of fundamental research into clinical application, and addresses pragmatic clinical challenges. Clinically, 3D printing has demonstrated its utility in precision and personalized medicine. Despite the rapid evolution of 3D printing technology, current constraints in printing apparatuses and the spectrum of printable bioinks preclude its routine clinical deployment. Thus, it is imperative to persist in refining printing techniques, discovering novel biomaterials, broadening printers’ biocompatibility with diverse biomaterials, and enhancing resolution. Concurrently, efforts must focus on augmenting cell viability and density, curtailing printing durations, expanding the dimensions of printed tissues, and reducing costs.

## Figures and Tables

**Table 1:** Characteristics of several bioprinting techniques.

Print Methods	Bioinks	Resolution	Material Deposition Rate	Suitability	References
**Laser-assisted** **printing**	Fibrinogen, collagen, GelMA	1–50 μm	High	High-resolution skin, vessel, and tumor models.	[62,63,64,65,66,67]
**Inkjet** **printing**	Collagen, poly(ethylene glycol) dimethacrylate (PEGDMA), fibrinogen, alginate, GelMA	50–500 μm	Medium	Medium-resolution structures; drug testing.	[13,68,69,70,71,72]
**Extrusion** **printing**	Gelatin, polycaprolactone (PCL), polyethylene glycol (PEG), alginate hyaluronic acid (HA), polyamide(PA), polydimethylsiloxane (PDMS) dECM, nanocellulose	>50 μm	Low	Large-scale tissue scaffolds.	[73,74,75,76,77,78,79,80,81,82]
**Photopolymerization**	Photosensitive Hydrogels	Sub 1 µm (TPP/DLP)	Medium to High	High-resolution, cell-laden tumor, and organ-on-chip models.	[83,84]

**Table 2:** Comparison of commonly used 3D bioprinting techniques.

Method	Advantages	Disadvantages	Latest Developments (2025)
**Inkjet Printing**	High efficiency; low cost; compatible with multi-material printing.	Limited viscosity of bioinks; potential cell damage from droplet ejection.	Advances in nozzle designs to reduce shear stress on cells.
**Extrusion Printing**	Affordable; wide range of bioink viscosities; high cell density deposition.	Low resolution; slower for complex structures; limited material types.	Multi-material extrusion allowing for gradient tissue constructs.
**Laser-Assisted Printing**	High precision; non-contact printing; adaptable for living cells.	Equipment cost; challenges with scalability; potential thermal effects.	LIFT techniques now employ hydrogel coatings instead of metal layers.
**Photopolymerization (TPP/DLP)**	Exceptional resolution (sub-micron); suitable for creating intricate structures.	Limited to photosensitive materials; potential phototoxicity.	Expanded use of biocompatible photoinitiators for living cell encapsulation.
